# Accuracy of Customized Prefabricated Screw-Type Immediate Provisional Restorations after Single-Implant Placement

**DOI:** 10.3390/jcm8040490

**Published:** 2019-04-11

**Authors:** Kyung Chul Oh, Jee-Hwan Kim, Chang-Woo Woo, Hong Seok Moon

**Affiliations:** 1 Department of Prosthodontics, Yonsei University College of Dentistry, Seoul 03722, Korea; kyungabc@yuhs.ac (K.C.O.); jee917@yuhs.ac (J.-H.K.); 2 Central Dental Laboratory, Yonsei University Dental Hospital, Seoul 03722, Korea; woochw@yuhs.ac

**Keywords:** single-implant, immediate provisionalization, digital workflow, computer-aided design, computer-assisted implant surgical guide, prosthetic-driven implant placement

## Abstract

Limited evidence is available comparing the differences between pre-operative and post-operative 3D implant positions from the viewpoint of prosthetics. We aimed to investigate the differences between preplanned positions of virtual provisional restorations and their actual positions following fully guided single-implant placement. Ten maxillary typodonts with missing right central incisors were imaged using cone-beam computed tomography, and digital impressions were obtained using an intraoral scanner. These data were imported into implant-planning software, following which the provisional restorations were designed. After data superimposition, an appropriate implant position was determined, and a computer-assisted implant surgical guide was designed for each typodont. Orders generated from the implant-planning software were imported into relevant computer-aided design software to design the custom abutments. The abutments, provisional restorations, and surgical guides were fabricated, and each restoration was cemented to the corresponding abutments, generating a screw-type immediate provisional restoration. The implants were placed using the surgical guides, and the screw-type provisional restorations were engaged to the implants. The typodonts were then rescanned using the intraoral scanner. The restorations designed at the treatment planning stage were compared with those in the post-operative scan using metrology software. The angular deviation around the central axis of the implant was measured, and the differences in the crown position were converted to root mean square (RMS) values. The post-operative provisional restorations exhibited an absolute angular deviation of 6.94 ± 5.78° and an RMS value of 85.8 ± 20.2 µm when compared with their positions in the pre-operative stage. Within the limitations of the present in vitro study, the results highlight the potential application of customized prefabricated immediate provisional restorations after single-implant placement.

## 1. Introduction

Digital dentistry has become a leading field in dental practice today and has taken dental implantology to a new level [1,2]. Radical improvements in the accuracy and resolution of cone-beam computed tomography (CBCT), intraoral, tabletop, and face scanners, the development of dental computer-aided design (CAD) software and computer-aided manufacturing (CAM) technology, and the application of additive manufacturing technology in the fabrication of computer-assisted implant surgical guides have all contributed to this revolution [3,4,5,6,7,8,9].

The digital workflow in implantology starts from data acquisition, for which CBCT and the scanners are used [9]. CBCT is used to visualize underlying hard tissue structures in three orthogonal (axial, sagittal, and coronal) planes, enabling assessment of available bone for dental implant placement [10]. Tabletop scanners enable indirect digitization of dental casts or impressions, whereas intraoral scanners directly digitize the clinical situation within the oral cavity at chairside [11,12,13]. Face scanners provide useful information regarding important facial references such as the interpupillary line, smile line, and facial midline for anterior-tooth rehabilitation [14]. The resultant data formats differ from each other: CBCT uses the Digital Imaging and Communications in Medicine (DICOM) format, while intraoral and tabletop scanners use the standard tessellation language (STL) format, and face scanners use the object code (OBJ) format [15].

Subsequently, these datasets are imported into the CAD software and implant-planning software to virtually design prostheses and determine the 3D position of implants [9]. This enables prediction of outcomes after the placement of definitive restoration, thereby facilitating effective communication with patients [7]. The last procedure is computer-assisted production by means of subtractive or additive manufacturing [16]. Implant abutments, prostheses, and surgical guides are fabricated using these methods [17].

It has long been accepted that implants should not be exposed to unintended force when placed in a partially edentulous area [18]. Hence, prostheses were not connected to implants during the healing period in these cases in the past, prolonging the interval between implant installation and a fixed-type prosthesis connection [19]. However, given the increasing esthetically driven demand to reduce the edentulous period, and with the development of implant surfaces, materials, components, and surgical and prosthetic protocols, implant-supported immediate provisionalization has been suggested as a viable option to maximize esthetic outcomes, particularly after single-implant placement [18,19,20,21,22,23,24,25,26]. These immediate provisional restorations can be provided either without any contacts in centric occlusion or during eccentric movements, or with functional loadings [27]. According to a recent consensus statement, it is recommended that no functional loadings be given to the restoration when rehabilitating a single edentulous maxillary anterior tooth with an implant and immediate provisional restoration [28].

Immediate provisionalization increases patient satisfaction and preserves gingival architecture around the implants [21,28]. However, patients are required to wait for an extended period of time at the clinic until provisional restorations are prepared from the final impressions obtained after implant placement or they must undergo intraoral relining procedures using the prefabricated provisional shells [25,29,30,31]. Not only do these methods require further steps, but there is a drawback in that the provisional restorations can only be fabricated in the finalized form after surgery is complete, because it was nearly impossible to place implants as planned without the technology available today. However, computer assistance has allowed for more accurate implant placement [32,33]. This guidance can be performed using either a static or dynamic approach [34].

Several studies have assessed the differences between pre-operative and post-operative three-dimensional (3D) implant positions [33,35,36,37]. However, no scientific evidence is available comparing these outcomes from the viewpoint of prosthetics. Hence, the present study aimed to compare the virtual crown-incorporated pre-operative data generated during the treatment planning stage with the post-operative scan data obtained after the immediate placement of a customized prefabricated screw-type provisional restoration, which was performed following single-implant placement using a fully guided surgery kit in a static approach. The potential sources of error in using the protocol of the present study were also discussed. The hypothesis was that customized prefabricated screw-type immediate provisional restorations are accurately connected to the implants in their predetermined positions after the placement of single implants using a complete-guidance implant surgery kit.

## 2. Experimental Section

**Figure 1:**
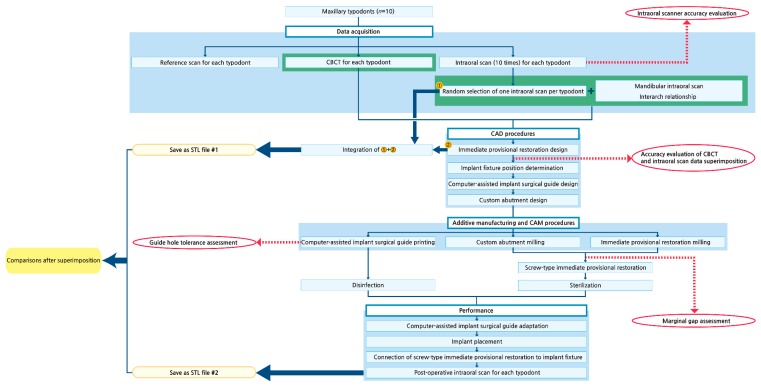
Schematic of the experimental protocols. CBCT, cone-beam computed tomography. CAD, computer-aided design. CAM, computer-aided manufacturing. STL, standard tessellation language.

The experimental protocols are summarized in Figure 1.

### 2.1. Modification of Dental Models

**Figure 2:**
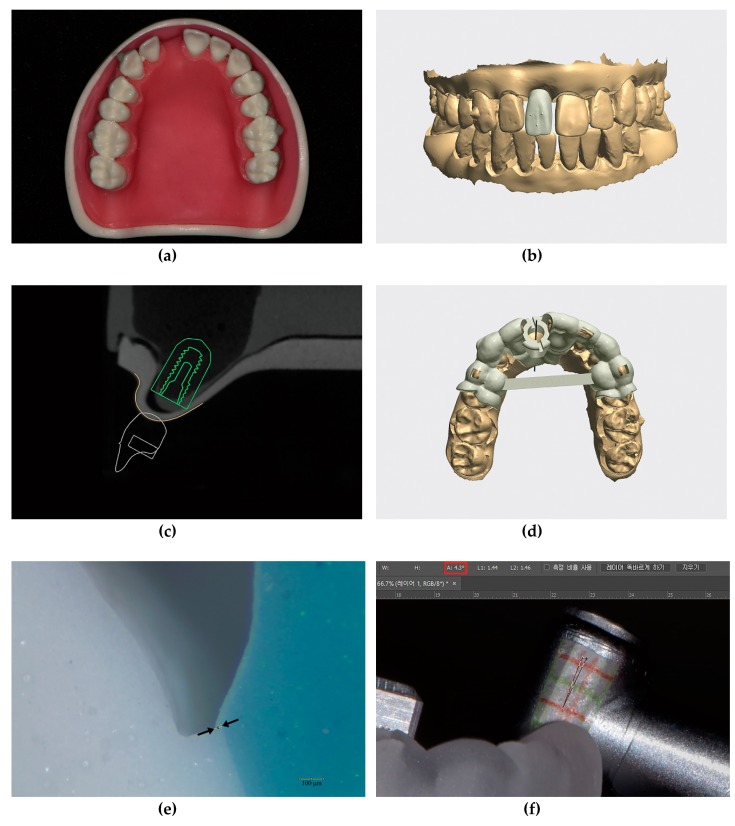

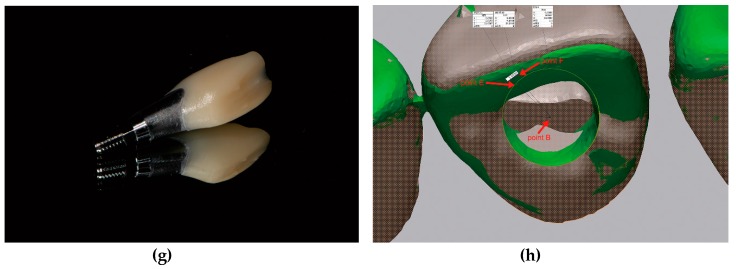
Details of the experimental protocols; (**a**) Maxillary typodont (CIBM01, Osstem, Busan, Korea) with a missing right central incisor; (**b**) Virtual single immediate provisional restoration design in the implant-planning software (Implant Studio version 2.17.1.4, 3Shape, Copenhagen, Denmark) showing three small projections on the labial surface and sufficient clearance from adjacent teeth; (**c**) Determination of implant fixture diameter, length (TS III 4.0 × 10 mm, Osstem, Busan, Korea), and implant position; (**d**) Metal-sleeveless computer-assisted implant surgical guide design for fully guided implant placement; (**e**) Sectioned silicone replica for evaluation of the marginal gap of the provisional restoration using a stereozoom microscope (SMZ168, Motic, Xiamen, China) at 45× magnification and image analysis software (ImageJ version 1.51k, National Institute of Health, Bethesda, MD, USA). The marginal gap was defined by the distance between the two black arrows; (**f**) Measurement of guide hole tolerance in the image-editing software (Photoshop 2018, Adobe, San Jose, CA, USA). The value marked with the red rectangle in the upper menu indicates the tolerance; (**g**) Customized prefabricated screw-type immediate provisional restoration; (**h**) An example image showing the angular deviation, which was defined by the angle formed by lines BE and BF. Point B, the reference point. Points E and F, points of crossing between the perpendicular lines (drawn from the centers of the triangles formed by the three small projections on the provisional restorations in each dataset) and the reference plane.

Ten typodonts (CIBM01, Osstem, Busan, Korea) with missing maxillary right central incisors were used for the present study. Radiopaque markers (Tetric N-Ceram, Ivoclar Vivadent, Schaan, Liechtenstein) were attached to the buccal surfaces of the maxillary first molars and to the labial surface of the maxillary right canine (Figure 2a).

### 2.2. Evaluation of Intraoral Scanner Accuracy (Trueness and Precision)

For each maxillary typodont, reference scan data were obtained using an industrial scanner (ATOS Triple scan, GOM, Braunschweig, Germany). An expert who is proficient in using this scanner provided technical assistance with obtaining the reference scan data. Then, 10 digital impressions were obtained for each maxillary arch using an intraoral scanner (Trios 3, 3Shape, Copenhagen, Denmark) by an experienced user (K.C.O.) to assess its accuracy. The scanners were calibrated before their use, and accuracy was assessed in accordance with previous research methods using metrology software (Geomagic Control X version 2018.0.1, 3D Systems, Rock Hill, SC, USA) [38,39]. Briefly, to evaluate the trueness of the intraoral scanner, the 10 intraoral scans obtained from each maxillary typodont were superimposed over their corresponding reference scan data one-by-one. To evaluate the precision of the intraoral scanner, the data from the ten intraoral scans obtained from each maxillary typodont were compared with one another, resulting in 45 sets of comparisons for each typodont. This procedure was repeated for the other nine maxillary typodonts. The resulting root mean square (RMS) values were used to represent trueness and precision, respectively.

### 2.3. Acquisition of Intraoral Scan Data

After confirming accuracy of the intraoral scanner, one maxillary digital impression was randomly selected for each typodont. For the selected maxillary digital impression, the mandibular digital impression and interarch relationship were additionally obtained using the same intraoral scanner.

### 2.4. Acquisition of CBCT Data

Each maxillary typodont was imaged using CBCT (Alphard 3030, Asahi Roentgen Ind., Co. Ltd., Kyoto, Japan) with the following parameters: 80 kVp and 8 mA over 17 s using a 100 × 100 mm field-of-view with a voxel size of 0.2 mm^3^.

### 2.5. CAD Procedures

Intraoral scan and CBCT data were imported into implant-planning software (Implant Studio version 2.17.1.4, 3Shape, Copenhagen, Denmark). A single-implant crown for an immediate provisional restoration was designed for the edentulous area, without providing any occlusal contacts with the opposing teeth. The design of the provisional restoration had the following specific features: three small projections added to the labial surface and sufficient clearance from the adjacent teeth (Figure 2b). The CBCT data were superimposed with intraoral scan data with respect to three radiopaque markers on the maxillary teeth. Appropriate implant fixture diameter, length (TS III 4.0 × 10 mm, Osstem, Busan, Korea), and position were determined based on the virtual crown arrangement (Figure 2c). Then, a metal-sleeveless computer-assisted implant surgical guide—for fully guided implant placement—was designed for each maxillary typodont (Figure 2d).

The orders generated by the software were imported into another CAD program (Dental System, 3Shape, Copenhagen, Denmark). A custom abutment was designed above the implant fixture, which automatically appeared in the software, as determined at the previous stages. The virtual implant crown also appeared in the same form as that in the implant-planning software. Few modifications were made to its contour; the margins of the virtual crown were adapted to the custom abutment, and a screw access hole was created to approach the abutment screw. Lastly, each maxillary arch, with its finalized crown arranged in position, was saved in the STL file format for future comparison.

### 2.6. Additive Manufacturing (3D Printing) and CAM Procedures

The implant surgical guides were fabricated using a polyjet 3D printer (Objet Eden260VS, Stratasys, Eden Prairie, MN, USA). The custom abutments were fabricated from a titanium alloy (Ti 6Al-4V ELI, Fort Wayne Metals, Fort Wayne, IN, USA) using a computer numerical control (CNC) automatic lathe (SR-20J type C, Star Micronics, Shizuoka, Japan). The provisional restorations were fabricated from a polymethylmethacrylate resin block (VIPI Block Trilux, VIPI Ltd., São Paulo, Brazil) using a five-axis milling machine (DWX-51D, Roland DG, Hamamatsu, Japan).

### 2.7. Evaluation of the Marginal Adaptation of the Provisional Restorations

All provisional restorations were evaluated for marginal adaptation on the corresponding custom abutments via a replica technique using vinyl polyether silicone material (Fit Checker Advanced, GC Corp, Tokyo, Japan) and vinyl polysiloxane materials (Aquasil Ultra LV, Dentsply Caulk, Milford, DE, USA; Exafine putty type, GC Corp, Tokyo, Japan) [40,41]. The thickness of the outermost vinyl polyether silicone material was measured at four points—labial, mesial, distal, and palatal—for every abutment/crown assembly, using a stereozoom microscope (SMZ168, Motic, Xiamen, China) at 45× magnification and image analysis software (ImageJ, version 1.51k, National Institute of Health, Bethesda, MD, USA) (Figure 2e).

### 2.8. Evaluation of the Guide Hole Tolerance of the Surgical Guides

Tolerance of the guide hole for each surgical guide was evaluated using a pair of photographs obtained after moving the hand-piece far labially and palatally, with the guide drill inserted [42]. The photographs were imported into the image-editing software (Photoshop 2018, Adobe, San Jose, CA, USA), and lines were drawn from each image by referring to the grid paper attached to the hand-piece. The angle formed by the two lines was defined as the tolerance (Figure 2f).

### 2.9. Preparation of Prefabricated Screw-Type Immediate Provisional Restorations

After evaluating the marginal adaptation, the provisional restorations were cemented to the corresponding abutments using resin cement (Multilink N, Ivoclar Vivadent, Schaan, Liechtenstein), producing screw-type immediate provisional restorations (Figure 2g).

### 2.10. Surgical and Prosthodontic Procedures: Implant Placement and Screw-Type Immediate Provisional Restoration Connection

The screw-type immediate provisional restorations were sterilized with ethylene oxide gas prior to surgery. The surgical guides were immersed in 0.12% chlorhexidine digluconate solution (Hexamedine, Bukwang, Seoul, Korea) for disinfection before implant placement. Then, the implant surgical guides were evaluated for accurate adaptation onto the typodonts. The implants were placed via a flapless technique using these guides and a complete-guidance kit (OneGuide, Osstem, Busan, Korea) by an experienced clinician (K.C.O.). Drilling was performed in accordance with the manufacturer’s recommendations. The prefabricated screw-type provisional restorations were engaged onto the implants immediately after implant placement using a torque wrench.

### 2.11. 3D Analysis of the Provisional Restoration Position

After connecting the restorations, the maxillary typodonts were scanned using the same intraoral scanner and strategies used for the pre-operative scans. The pre-operative STL data, with its finalized crown arranged in position on the maxillary arch, served as the reference data, and the post-operative scan data were superimposed over these data using the same metrology software previously used.

First, the angular deviations between the crowns in the post-operative scan data and those at the treatment planning stage were evaluated for each typodont. The specific reference plane (plane A) was defined by selecting the bilateral mesiobuccal cusps of the maxillary first molars and the incisal edge of the maxillary left central incisor. Then, the reference point (point B) was defined as the point of crossing between the imaginary long axis of the screw access hole and plane A. Subsequently, two points (points C and D) were defined as the centers of the triangles, which were formed by connecting the three small projections on the labial surfaces of the provisional restorations from each pre-operative and post-operative dataset, respectively. Perpendicular lines were drawn from these points to plane A individually, creating the two corresponding points (points E and F) on the plane. The angular deviations were defined by the angle formed by lines BE and BF (Figure 2h). When viewed from the occlusal aspect, if the post-operative provisional restoration was rotated in a clockwise direction, compared to the pre-operative STL data, the angular deviation was denoted with a plus sign; whereas, if it was rotated in a counterclockwise direction, the angular deviation was denoted with a minus sign. However, when calculating the mean and standard deviation, the minus signs were converted to plus signs to obtain absolute values for each.

Following this, the pre-operative and post-operative STL files were trimmed, leaving six maxillary anterior teeth for each file. The RMS values were calculated for each typodont to compare the 3D positions of the provisional restorations at the treatment planning and post-operative stages.

### 2.12. Statistical Analysis

As the present study did not include a control group, we only reported descriptive data. The means ± standard deviations for each dataset were summarized using statistical software (IBM SPSS Statistics version 23.0, IBM Corp, Somers, NY, USA).

## 3. Results

The trueness and precision of the intraoral scanner were 153.8 ± 23.0 µm and 69.5 ± 14.3 µm, respectively (Table A1 and Table A2, respectively). The marginal gap of the provisional restorations was 87.8 ± 17.2 µm (Table A3). All 10 surgical implant guides were stable on the typodonts and exhibited accurate adaptation. The guide hole tolerance was 4.3 ± 0.9° (Table A4).

**Figure 3:**
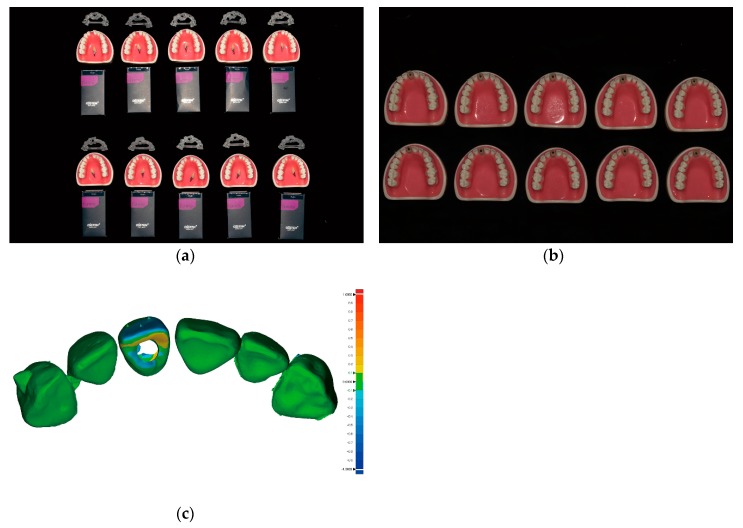
Comparison of the post-operative immediate provisional restoration position with the predesigned restoration position; (**a**) Ten sets of preparations prior to implant placement arranged in two rows (from top to bottom within each row) as follows: implant surgical guides, typodonts and customized prefabricated screw-type immediate provisional restorations placed above the typodonts, and implant fixtures; (**b**) Ten maxillary typodonts after the placement of implants and screw-type immediate provisional restorations; (**c**) An example image showing the difference between the pre-operative data and the post-operative scan data using metrology software (Geomagic Control X version 2018.0.1, 3D Systems, Cary, NC, USA). The critical value was set at ±1000 µm, and the tolerance value was set at ± 100 µm.

All implants were fixed firmly into the simulated alveolar bone structure within the typodonts. The absolute angular deviation between the pre-operative data (i.e., the maxillary arches with their finalized crowns arranged in position at the treatment planning stage) and the post-operative scan data was 6.94 ± 5.78°, and the RMS value was 85.8 ± 20.2 µm (Figure 3 and Table 1).

## 4. Discussion

As digital technology penetrates rapidly into the dental field, we are now beginning to accomplish procedures previously thought impossible. To our knowledge, the present study is the first to report the accuracy of customized prefabricated single-implant provisional restorations placed immediately after prosthetic-driven implant placement. Because there is currently no available data regarding this theme, we did not aim to compare the accuracy among different implant systems; rather, we aimed to present the quantitative measurements obtained from our experiments.

We first evaluated the accuracy of the intraoral scanner. The scanner that was shown to provide the best combination of speed, trueness, and precision for complete-arch scanning was selected [38]. Scanning strategies were unified throughout the study to avoid the influence of different scanning strategies on the results [43]. We then compared our accuracy data with those obtained by previous studies involving similar conditions (full-arch scanning of the typodont using the same intraoral scanner or full-arch scanning of the typodont using RMS values as the reference standard) [38,44]. Based on the RMS values for trueness and precision, we concluded that the data obtained using the intraoral scanner in our study were valid. While scanning the maxillary anterior sextant only may have increased the accuracy of the scanned data, we obtained full-arch scans, as regular check-up-based full-arch scans may aid in identifying important changes within the oral cavity.

To facilitate the superimposition of CBCT and intraoral scan data, radiopaque markers were attached to the three teeth. The sites of attachment were selected so that they were widely distributed in a triangular fashion, to achieve more accurate superimposition. The accuracy of the superimposition was verified by referring to the color map that appears within the software; the tolerance value was set to ±200 µm. Superimpositions within this range appeared in green. If green shading dominated more than 90% of the total superimposed 3D image, the superimposition was accepted as accurate. The voxel size of the CBCT was set to 0.2 mm^3^ to achieve higher image quality and resolution [45].

The provisional restorations were milled using a five-axis milling machine to achieve high accuracy [46]. The supports for the provisional restorations were attached at the apical portion of the restorations, since this portion was not to be compared with their initial design. No further adjustments were conducted on the provisional restorations, except for support removal. For the fabrication of custom abutments, a CNC automatic lathe was used to ensure the accurate reflection of their CAD designs. The implant surgical guides were fabricated by the manufacturer of the implant, since the implant company fabricates the guides using 3D printers that adopt polyjet technology, which is known for its high accuracy [47].

Proper seating of the restorations onto the custom abutments was verified to exclude the influence of improper seating on our results. A marginal discrepancy of approximately 90 µm was considered acceptable from a clinical perspective [48,49]. Dental cement was applied minimally to eliminate errors caused by the unintended attachment of excessive cement, which may leak through the screw access hole. The guide hole tolerance of the surgical guides was similar to that measured in surgical guides in previous studies [42,50,51].

Considering that healing abutments are connected to implants in a sterilized state in conventional protocols, it is important that the provisional restorations be sterile. Our novel approach enables the screw-type provisional restoration to be in prepared in a sterilized state, since it is prepared prior to surgery. Ethylene oxide gas sterilization was selected since it does not alter material properties, as opposed to autoclave sterilization [52]. For the disinfection of the surgical guides, 0.12% chlorhexidine digluconate solution was selected, per the manufacturer’s guidelines. Surgical guides were checked for their adaptation. Since physical gypsum models were not fabricated, the guides cannot be assessed until patient visits in actual clinical situations. Hence, we evaluated the adaptation of surgical guides from a clinical perspective, using the windows on the guides and by verifying the absence of mobility.

Each guide drill was used until the guide drill stopper made contact with the guide hole sleeve. The implant was installed until the top level of the fixture driver matched with that of the guide hole sleeve and the yellow marking on the fixture driver matched with the slot of the guide hole sleeve. Since the location of the top surface of the fixture driver level and the yellow marking on the fixture driver may appear to differ depending on the direction from which it is viewed, we aimed to examine the correspondence between the two from a vertical (90°) perspective in order to ensure the appropriate depth and hexagonal position of the implants. In addition, we performed several preliminary drilling trials to gauge differences in drilling the simulated bone structures using spare typodonts.

The restorations were designed to be free of contacts with adjacent teeth to eliminate the possibility of incomplete connection to implants due to premature contact with adjacent teeth. After connecting the screw-type restoration, the maxillary typodont was scanned using the same intraoral scanner, since its superior precision had been confirmed during the pre-operative evaluation stage. To eliminate inter-operator variability, a single experienced user of this scanner performed all scans and surgical/prosthodontic procedures.

For measurements of angular deviation, we aimed to determine how much the provisional restoration had rotated along its rotational axis from the occlusal point of view. Hence, a reference plane (plane A) resembling the occlusal plane was first defined, and the reference point (point B) was defined as the point at which plane A and the rotational axis of the implant crossed. This point served as the single reference point for measurements of angular deviations. Then, the angular deviation between the pre-operative STL data and post-operative scan data was measured on the reference plane.

RMS values may differ based on the regions of interest evaluated. For consistency, we restricted the regions of interest to the tooth portion throughout the superimposition procedures. However, gingival portions were included for the edentulous areas of the maxillary right central incisors when evaluating the accuracy of the intraoral scanner. Moreover, only six maxillary anterior teeth were included in the measurement of RMS values between the pre-operative and post-operative STL files, since full-arch scan data may include errors associated with the inaccuracy inherent to the intraoral scanner. In the present study, we obtained small angular deviation and RMS values, suggestive of a bright future for the procedures investigated. Our findings suggest that all preparations for the placement of an immediate provisional restoration following single-implant placement can be made prior to surgery, with minimal manipulation at chairside.

In addition to providing quantitative insight into the outcomes of our procedures, our findings also highlight how the final results will look following an immediate restoration placement. It is difficult to visualize how the prosthesis will look based on information regarding fixture deviations alone, as the implant fixtures are embedded within the bone. However, visualization of the prosthodontic structures enables a more intuitive understanding of the results at first glance.

Potential sources of error during the present protocol and the efforts made to minimize them are summarized in Table 2. Errors due to the inherent nature of an in vitro study design have been excluded from consideration (e.g., absence of patient movement, absence of saliva, differences in bone quality/quantity, differences in refractive indices and radiopacity when compared with natural teeth and gingival tissues). Various software programs and equipment types are available for digital dentistry. Even when using the same software, different versions may yield different results. Hence, our findings should be interpreted with caution.

The genuine digital revolution of single-implant treatment will be possible when the customized components can be prepared prior to the implant surgery and fit accurately at the intended positions after surgery. Currently, there are increasing numbers of commercially available implant systems that enable fully guided implant placement. Comparative analyses with different fully guided surgery kits are mandatory to investigate if accuracy differs among them. Moreover, further clinical studies are necessary to elucidate the clinical effectiveness of the presented protocol.

## 5. Conclusions

Within the limitations of the present in vitro study, the results highlight feasibility of preparing a customized prefabricated screw-type immediate provisional restoration prior to single-implant placement in esthetic regions. Although the clinically acceptable angular deviation and RMS values between the pre-operative and post-operative prefabricated immediate provisional restorations require further exploration, the restoration deviated in amounts that are hardly detectable from a clinical viewpoint, thereby requiring minimal adjustment, if any, at chairside. The workflow will not only increase the efficiency of the treatments, but will also improve patient satisfaction, because it enables provisional restorations to be ready-made at any time when patients hope to visit, provided that digital full-arch scans have been recently obtained. Potential sources of error during the whole procedure including data acquisition, processing, and production should be taken into consideration for the clinical application of the present protocol.

## Figures and Tables

**Table 1:** The angular deviation and root mean square (RMS) value between the pre-operative data and the post-operative scan data.

	MOD#1	MOD#2	MOD#3	MOD#4	MOD#5	MOD#6	MOD#7	MOD#8	MOD#9	MOD#10	Mean ± SD
**Angular deviation (°)**	−5.89	−0.05	−9.07	−11.87	5.71	−12.49	−1.17	17.90	3.03	2.24	6.94 ± 5.78 (Absolute values)
**RMS (µm)**	60.8	53.4	111.7	99.5	87.3	80.7	104.8	103.1	91.1	65.3	85.8 ± 20.2

A plus sign in the angular deviation indicates that the post-operative provisional restoration is rotated in a clockwise direction, compared to the pre-operative STL data when viewed from the occlusal aspect; whereas, a minus sign in the angular deviation indicates that the post-operative provisional restoration is rotated in a counterclockwise direction. However, the mean and standard deviation of the angular deviations are calculated in terms of absolute values. MOD, model. SD, standard deviation. RMS, root mean square. STL, standard tessellation language.

**Table 2:** Potential sources of error and the efforts to minimize them. CBCT, cone-beam computed tomography.

Procedures		Factors That May Contribute to Error Reduction	Efforts to Reduce Errors	Methods to Confirm That Potential Sources of Error Had Been Minimized
**Data acquisition**	Intraoral scanning	Calibration procedures	Calibrated the intraoral scanner before obtaining the data	By assessing the accuracy (trueness and precision) of the intraoral scanner
		Scanning strategy	Unified scanning strategy	
	CBCT	Calibration procedures	Calibrated the CBCT scanner before obtaining the data	
		Voxel size	Applied voxel size of 0.2 mm^3^	
**Data processing**	Superimposition (CBCT and intraoral scan data)	Careful selection of three pairwise points	Referred to the color maps within the software	By confirming that the color maps were mostly within the range of ± 200 µm
**Data production**	Computer-assisted implant surgical guides	Selection of additive manufacturing principles	Used a polyjet 3D printer	
	Custom abutments and immediate provisional restorations	Selection of milling machine	Used a five-axis milling machine for the fabrication of provisional restorations	
		Proper adaptation between the two	Used a computer numerical control automatic lathe for the fabrication of custom abutments	By evaluating the marginal gap
		Appropriate amount of cement use	Used a minimal amount of dental cement	
		Selection of proper support position	Determined the appropriate support attachment position for provisional restorations	
**Pre-operative preparation**	Sterilization/Disinfection	Selection of appropriate sterilization/disinfection methods	Sterilized with ethylene oxide gas for the screw-type immediate provisional restorations	
			Disinfected with 0.12% chlorhexidine digluconate solution for the surgical guides	
**Performance**	Placement of the implant surgical guides	Adaptation onto the typodonts	Created windows on the guide in the planning stage	By inspecting the adaptation via windows and absence of guide movement
		Guide hole tolerance	Used a polyjet 3D printer	By evaluating the guide hole tolerance
	Surgical procedures	Angle at which the fixture driver is viewed	Tried to view the fixture driver from a vertical perspective	
		Bony imitation structure of the typodonts	Conducted a pilot study to gauge differences between typodont structures and real bone in clinical settings	
	Prosthodontic procedures	Amount of torque applied	Provided consistent torque throughout the study with the torque gauge	By confirming the values on the torque gauge
		Improper seating of the screw-type provisional restorations into the fixtures due to the tight interproximal contacts	Designed the restoration to be free of contacts with adjacent teeth	By confirming the absence of interproximal contacts after connecting the prosthesis
	Post-operative intraoral scanning	Calibration procedures	Calibrated the scanner before its use	Not applicable: had been confirmed during the pre-operative evaluation stage
		Scanning strategy	Applied the same scanning protocols as in the pre-operative scanning	
**Final verification**	Superimposition	Regions of interest	Regions of interest were restricted to the tooth portion	
**Overall consideration**		Inter-operator variability	All scans and surgical/prosthodontic procedures performed by a single experienced clinician	

## Appendix A

**Table A1:** Trueness of full-arch scan performed on each maxillary typodont with an intraoral scanner (Trios 3, 3Shape, Copenhagen, Denmark) expressed in terms of the root mean square (RMS) values (µm). MOD, model. SD, standard deviation.

	MOD#1	MOD#2	MOD#3	MOD#4	MOD#5	MOD#6	MOD#7	MOD#8	MOD#9	MOD#10	Overall Mean ± SD
	133.7	147.3	161.4	107.1	154.7	149.8	143.7	120.1	189.6	181.7	
	138.0	137.6	153.4	117.0	158.6	163.3	135.8	112.3	179.1	171.9	
	159.7	241.8	176.1	117.3	166.7	149.3	135.3	114.3	181.1	188.8	
	134.4	145.8	160.9	126.6	149.6	158.6	139.2	104.1	205.6	184.6	
	135.9	136.2	137.8	171.2	157.8	149.0	142.2	119.5	189.7	196.0	
	140.7	147.5	151.8	125.4	166.5	147.3	142.0	113.3	181.6	191.9	
	168.7	165.0	161.6	128.8	162.2	160.0	127.2	104.8	176.7	177.1	
	137.8	181.2	156.7	116.7	162.9	150.8	147.9	124.4	179.5	175.4	
	138.4	147.1	155.3	139.7	255.0	195.4	150.0	118.6	192.6	168.9	
	141.3	146.6	152.7	123.1	192.0	149.6	133.5	115.7	188.1	171.4	
**Mean**	142.9	159.6	156.8	127.3	172.6	157.3	139.7	114.7	186.4	180.8	153.8 ± 23.0

**Table A2:** Precision of full-arch scan performed on each maxillary typodont with an intraoral scanner (Trios 3, 3Shape, Copenhagen, Denmark) expressed in terms of the root mean square (RMS) values (µm). MOD, model. SD, standard deviation.

	MOD#1	MOD#2	MOD#3	MOD#4	MOD#5	MOD#6	MOD#7	MOD#8	MOD#9	MOD#10	Overall Mean ± SD
	29.9	92.3	46.7	42.5	83.2	63.7	37.9	109.6	101.1	68.9	
	115.8	327.6	146.2	32.9	86.6	61.0	85.7	35.2	52.2	31.7	
	59.0	117.1	34.8	50.0	37.0	58.8	61.2	61.7	135.9	108.3	
	50.5	138.0	38.7	131.5	40.7	75.3	83.6	36.5	82.3	31.1	
	63.2	158.0	70.2	58.6	73.2	50.0	38.9	40.7	58.5	37.4	
	119.4	180.3	124.0	61.3	55.7	69.4	48.5	68.8	52.4	33.7	
	46.9	250.2	70.3	35.0	59.6	55.6	42.3	39.9	91.2	38.8	
	59.7	179.9	76.1	98.8	169.8	118.5	121.8	117.7	38.7	79.7	
	59.0	144.9	28.9	51.3	125.1	52.8	65.7	123.5	108.4	46.5	
	127.9	239.6	93.6	30.7	32.5	53.0	85.1	106.3	46.1	55.9	
	57.9	39.0	71.1	46.2	52.3	61.5	56.9	56.3	74.4	37.5	
	49.2	45.5	40.6	139.1	57.5	88.4	89.9	120.4	40.0	75.6	
	71.2	117.1	38.1	56.0	25.3	47.9	30.0	87.6	44.5	84.4	
	143.3	137.4	97.1	81.9	30.2	40.6	41.9	41.7	52.1	41.0	
	45.5	143.9	35.4	42.2	28.4	43.2	45.4	108.3	32.1	37.4	
	55.8	125.1	46.1	104.0	105.6	68.4	106.5	31.0	82.0	20.5	
	57.5	96.5	37.0	50.3	50.2	85.0	61.5	35.1	49.6	27.0	
	65.3	209.0	166.0	41.6	59.0	32.4	38.2	49.4	133.6	94.6	
	76.7	175.4	98.3	139.1	67.2	41.8	23.8	26.6	50.0	33.1	
	53.2	103.8	86.2	54.4	36.8	22.8	90.2	31.1	36.3	55.3	
	41.2	63.2	33.6	62.3	32.6	31.2	50.4	57.5	24.7	26.1	
	72.0	66.2	72.3	30.5	36.4	26.9	105.2	42.0	49.9	28.3	
	65.6	104.4	75.2	102.1	101.3	102.4	23.8	112.8	49.3	64.2	
	51.1	115.2	148.0	47.4	47.4	42.3	33.7	110.2	118.5	37.3	
	31.2	47.2	48.3	94.5	23.9	29.5	34.5	69.4	76.0	103.9	
	28.1	98.5	96.4	25.3	49.7	34.9	52.7	35.9	93.8	107.9	
	91.0	127.4	140.8	101.4	41.1	53.6	25.9	26.8	113.6	77.9	
	33.5	139.1	93.1	52.6	39.9	44.4	77.3	89.1	81.1	63.3	
	28.5	117.9	91.2	57.1	186.9	131.9	46.9	59.8	131.8	37.1	
	25.8	88.2	45.4	24.9	122.9	21.7	24.1	53.5	30.3	55.3	
	39.7	62.7	52.9	107.8	55.0	50.0	91.6	34.9	27.1	28.4	
	106.6	92.3	106.2	217.9	38.9	66.3	49.9	74.7	37.0	47.5	
	27.8	87.1	52.9	158.9	40.8	59.7	105.0	34.8	23.0	55.9	
	35.9	69.8	57.3	50.9	186.5	151.5	24.1	135.5	68.4	97.8	
	37.8	52.3	42.3	98.2	118.7	28.4	35.4	130.0	60.5	67.0	
	72.8	42.3	58.1	122.9	29.5	33.7	30.1	46.4	26.5	54.6	
	32.6	44.9	30.8	75.9	25.8	30.1	46.7	54.1	45.4	69.8	
	30.3	34.6	31.1	54.5	116.2	102.8	100.8	105.8	59.1	115.7	
	27.5	36.3	62.3	29.0	53.3	48.1	55.8	100.1	79.1	75.6	
	100.3	24.1	58.2	58.7	21.1	31.1	65.9	96.2	48.2	26.5	
	91.5	42.0	53.5	164.1	121.0	78.2	71.7	52.9	52.4	49.1	
	79.4	44.7	123.4	106.4	67.4	69.0	31.5	65.2	77.4	23.0	
	28.6	46.1	33.6	97.5	127.2	94.8	161.5	155.6	78.7	50.7	
	30.4	45.4	59.9	55.2	67.0	56.2	81.7	161.3	56.3	30.6	
	22.8	35.2	71.8	59.1	52.3	143.3	39.1	26.0	173.4	34.5	
**Mean**	58.6	105.5	70.8	75.6	68.4	61.2	60.5	72.4	67.6	54.8	69.5 ± 14.3

**Table A3:** Marginal gap (µm) of the immediate provisional restorations measured by replica technique. MOD, model. SD, standard deviation.

	MOD#1	MOD#2	MOD#3	MOD#4	MOD#5	MOD#6	MOD#7	MOD#8	MOD#9	MOD#10	Overall Mean ± SD
**Labial**	84.1	102.6	54.6	81.4	62.5	74.6	142.2	86.1	111.4	66.1	
**Mesial**	88.6	80.3	99.5	32.0	66.6	151.9	135.3	92.8	71.6	87.1	
**Distal**	32.3	88.6	70.4	85.6	106.9	78.0	102.1	63.1	67.6	112.9	
**Palatal**	43.9	70.4	156.0	53.5	83.6	119.5	93.3	131.9	95.2	87.2	
**Mean**	62.2	85.5	95.1	63.1	79.9	106	118.2	93.5	86.5	88.3	87.8 ± 17.2

**Table A4:** Guide hole tolerance (°) of the computer-assisted implant surgical guides. MOD, model. SD, standard deviation.

	MOD#1	MOD#2	MOD#3	MOD#4	MOD#5	MOD#6	MOD#7	MOD#8	MOD#9	MOD#10	Mean ± SD
**Tolerance**	4.3	3.1	4.2	4.7	5.0	5.5	3.0	3.6	5.6	4.3	4.3 ± 0.9

## References

[1] Ludlow M., Renne W. (2017). Digital workflow in implant dentistry. Curr. Oral Health Rep..

[2] Van Noort R. (2012). The future of dental devices is digital. Dent. Mater..

[3] Barazanchi A., Li K.C., Al-Amleh B., Lyons K., Waddell J.N. (2017). Additive technology: Update on current materials and applications in dentistry. J. Prosthodont..

[4] Block M.S. (2018). Dental implants: The last 100 years. J. Oral Maxillofac. Surg..

[5] Patel N. (2010). Integrating three-dimensional digital technologies for comprehensive implant dentistry. J. Am. Dent. Assoc..

[6] Zimmermann M., Mehl A., Mormann W.H., Reich S. (2015). Intraoral scanning systems—A current overview. Int. J. Comput. Dent..

[7] Joda T., Gallucci G.O. (2015). The virtual patient in dental medicine. Clin. Oral Implants Res..

[8] Joda T., Wolfart S., Reich S., Zitzmann N.U. (2018). Virtual dental patient: How long until it’s here?. Curr. Oral Health Rep..

[9] Mangano F., Shibli J.A., Fortin T. (2016). Digital dentistry: New materials and techniques. Int. J. Dent..

[10] Scarfe W.C., Farman A.G. (2008). What is cone-beam CT and how does it work?. Dent. Clin. North Am..

[11] Wesemann C., Muallah J., Mah J., Bumann A. (2017). Accuracy and efficiency of full-arch digitalization and 3d printing: A comparison between desktop model scanners, an intraoral scanner, a CBCT model scan, and stereolithographic 3D printing. Quintessence Int..

[12] Vecsei B., Joos-Kovacs G., Borbely J., Hermann P. (2017). Comparison of the accuracy of direct and indirect three-dimensional digitizing processes for CAD/CAM systems—An in vitro study. J. Prosthodontics Res..

[13] Kirschneck C., Kamuf B., Putsch C., Chhatwani S., Bizhang M., Danesh G. (2018). Conformity, reliability and validity of digital dental models created by clinical intraoral scanning and extraoral plaster model digitization workflows. Comput. Biol. Med..

[14] Sancho-Puchades M., Fehmer V., Hammerle C., Sailer I. (2015). Advanced smile diagnostics using CAD/CAM mock-ups. Int. J. Esthet. Dent..

[15] Mangano C., Luongo F., Migliario M., Mortellaro C., Mangano F.G. (2018). Combining intraoral scans, cone beam computed tomography and face scans: The virtual patient. J. Craniofac. Surg..

[16] Joda T., Zarone F., Ferrari M. (2017). The complete digital workflow in fixed prosthodontics: A systematic review. BMC Oral Health.

[17] Neugebauer J., Stachulla G., Ritter L., Dreiseidler T., Mischkowski R.A., Keeve E., Zoller J.E. (2010). Computer-aided manufacturing technologies for guided implant placement. Expert Rev. Med. Devices.

[18] Buser D., Sennerby L., De Bruyn H. (2017). Modern implant dentistry based on osseointegration: 50 years of progress, current trends and open questions. Periodontol. 2000.

[19] Siadat H., Alikhasi M., Beyabanaki E. (2017). Interim prosthesis options for dental implants. J. Prosthodont..

[20] Kan J.Y.K., Rungcharassaeng K., Deflorian M., Weinstein T., Wang H.L., Testori T. (2018). Immediate implant placement and provisionalization of maxillary anterior single implants. Periodontol. 2000.

[21] Ganeles J., Wismeijer D. (2004). Early and immediately restored and loaded dental implants for single-tooth and partial-arch applications. Int. J. Oral Maxillofac. Implants.

[22] Kupeyan H.K., May K.B. (1998). Implant and provisional crown placement: A one stage protocol. Implant Dent..

[23] Wohrle P.S. (1998). Single-tooth replacement in the aesthetic zone with immediate provisionalization: Fourteen consecutive case reports. Pract. Periodontics Aesthet. Dent..

[24] Touati B., Guez G. (2002). Immediate implantation with provisionalization: From literature to clinical implications. Pract. Proced Aesthet Dent.

[25] Kan J.Y., Rungcharassaeng K., Lozada J. (2003). Immediate placement and provisionalization of maxillary anterior single implants: 1-year prospective study. Int. J. Oral Maxillofac. Implants.

[26] Cooper L.F., Reside G.J., Raes F., Garriga J.S., Tarrida L.G., Wiltfang J., Kern M., De Bruyn H. (2014). Immediate provisionalization of dental implants placed in healed alveolar ridges and extraction sockets: A 5-year prospective evaluation. Int. J. Oral Maxillofac. Implants.

[27] Cochran D.L., Morton D., Weber H.P. (2004). Consensus statements and recommended clinical procedures regarding loading protocols for endosseous dental implants. Int. J. Oral Maxillofac. Implants.

[28] Feine J., Abou-Ayash S., Al Mardini M., de Santana R.B., Bjelke-Holtermann T., Bornstein M.M., Braegger U., Cao O., Cordaro L., Eycken D. (2018). Group 3 ITI consensus report: Patient-reported outcome measures associated with implant dentistry. Clin. Oral Implants Res..

[29] Zuiderveld E.G., Meijer H.J.A., den Hartog L., Vissink A., Raghoebar G.M. (2018). Effect of connective tissue grafting on peri-implant tissue in single immediate implant sites: A rct. J. Clin. Periodontol..

[30] Oh K.C., Jeon C., Park J.M., Shim J.S. (2019). Digital workflow to provide an immediate interim restoration after single-implant placement by using a surgical guide and a matrix-positioning device. J. Prosthet. Dent..

[31] Hui E., Chow J., Li D., Liu J., Wat P., Law H. (2001). Immediate provisional for single-tooth implant replacement with branemark system: Preliminary report. Clin. Implant Dent. Relat. Res..

[32] Raico Gallardo Y.N., da Silva-Olivio I.R.T., Mukai E., Morimoto S., Sesma N., Cordaro L. (2017). Accuracy comparison of guided surgery for dental implants according to the tissue of support: A systematic review and meta-analysis. Clin. Oral Implants Res..

[33] Nickenig H.J., Wichmann M., Hamel J., Schlegel K.A., Eitner S. (2010). Evaluation of the difference in accuracy between implant placement by virtual planning data and surgical guide templates versus the conventional free-hand method—A combined in vivo–in vitro technique using cone-beam CT (Part II). J. Craniomaxillofac. Surg..

[34] D’Haese J., Ackhurst J., Wismeijer D., De Bruyn H., Tahmaseb A. (2017). Current state of the art of computer-guided implant surgery. Periodontol. 2000.

[35] Van Assche N., van Steenberghe D., Guerrero M.E., Hirsch E., Schutyser F., Quirynen M., Jacobs R. (2007). Accuracy of implant placement based on pre-surgical planning of three-dimensional cone-beam images: A pilot study. J. Clin. Periodontol..

[36] Younes F., Cosyn J., De Bruyckere T., Cleymaet R., Bouckaert E., Eghbali A. (2018). A randomized controlled study on the accuracy of free-handed, pilot-drill guided and fully guided implant surgery in partially edentulous patients. J. Clin. Periodontol..

[37] Vermeulen J. (2017). The accuracy of implant placement by experienced surgeons: Guided vs freehand approach in a simulated plastic model. Int. J. Oral Maxillofac. Implants.

[38] Renne W., Ludlow M., Fryml J., Schurch Z., Mennito A., Kessler R., Lauer A. (2017). Evaluation of the accuracy of 7 digital scanners: An in vitro analysis based on 3-dimensional comparisons. J. Prosthet. Dent..

[39] Rehmann P., Sichwardt V., Wostmann B. (2017). Intraoral scanning systems: Need for maintenance. Int. J. Prosthodontics.

[40] Huang Z., Zhang L., Zhu J., Zhang X. (2015). Clinical marginal and internal fit of metal ceramic crowns fabricated with a selective laser melting technology. J. Prosthet. Dent..

[41] Reich S., Wichmann M., Nkenke E., Proeschel P. (2005). Clinical fit of all-ceramic three-unit fixed partial dentures, generated with three different CAD/CAM systems. Eur. J. Oral Sci..

[42] Oh K.C., Park J.M., Shim J.S., Kim J.H., Kim J.E., Kim J.H. (2019). Assessment of metal sleeve-free 3d-printed implant surgical guides. Dent. Mater..

[43] Muller P., Ender A., Joda T., Katsoulis J. (2016). Impact of digital intraoral scan strategies on the impression accuracy using the trios pod scanner. Quintessence Int..

[44] Jeong I.D., Lee J.J., Jeon J.H., Kim J.H., Kim H.Y., Kim W.C. (2016). Accuracy of complete-arch model using an intraoral video scanner: An in vitro study. J. Prosthet. Dent..

[45] Fokas G., Vaughn V.M., Scarfe W.C., Bornstein M.M. (2018). Accuracy of linear measurements on CBCT images related to presurgical implant treatment planning: A systematic review. Clin. Oral Implants Res..

[46] Abduo J., Lyons K., Bennamoun M. (2014). Trends in computer-aided manufacturing in prosthodontics: A review of the available streams. Int. J. Dent..

[47] Kim S.Y., Shin Y.S., Jung H.D., Hwang C.J., Baik H.S., Cha J.Y. (2018). Precision and trueness of dental models manufactured with different 3-dimensional printing techniques. Am. J. Orthod. Dentofac. Orthop..

[48] McLean J.W., von Fraunhofer J.A. (1971). The estimation of cement film thickness by an in vivo technique. Br. Dent. J..

[49] Kelvin Khng K.Y., Ettinger R.L., Armstrong S.R., Lindquist T., Gratton D.G., Qian F. (2016). In vitro evaluation of the marginal integrity of CAD/CAM interim crowns. J. Prosthet. Dent..

[50] Van Assche N., Quirynen M. (2010). Tolerance within a surgical guide. Clin. Oral Implants Res..

[51] Koop R., Vercruyssen M., Vermeulen K., Quirynen M. (2013). Tolerance within the sleeve inserts of different surgical guides for guided implant surgery. Clin. Oral Implants Res..

[52] Munker T., van de Vijfeijken S., Mulder C.S., Vespasiano V., Becking A.G., Kleverlaan C.J., Becking A.G., Dubois L., Karssemakers L.H.E., Milstein D.M.J. (2018). Effects of sterilization on the mechanical properties of poly (methyl methacrylate) based personalized medical devices. J. Mech. Behav. Biomed. Mater..

